# Airborne Influenza Virus in Daycare Centers

**DOI:** 10.3390/v16060822

**Published:** 2024-05-22

**Authors:** Jia Lin Zhang, Yu-Chun Wang, Yi Lien Lee, Chun-Yuh Yang, Pei-Shih Chen

**Affiliations:** 1 Department of Public Health, College of Health Science, Kaohsiung Medical University, Kaohsiung 80708, Taiwan; cindy3464919@gmail.com (J.L.Z.);; 2 Department of Environmental Engineering, College of Engineering, Chung Yuan Christian University, Taoyuan 32023, Taiwan; ycwang@cycu.edu.tw; 3 Soil and Groundwater Remediation Division, CPC Corporation, Kaohsiung 811251, Taiwan; 4 Institute of Environmental Engineering, College of Engineering, National Sun Yat-Sen University, Kaohsiung 804201, Taiwan; 5 Department of Medical Research, Kaohsiung Medical University Hospital, Kaohsiung 80756, Taiwan; 6 Research Center for Precision Environmental Medicine, Kaohsiung Medical University, Kaohsiung 80708, Taiwan; 7 Institute of Wildlife Conservation, College of Veterinary Medicine, National Pingtung University of Science and Technology, Pingtung 91201, Taiwan

**Keywords:** daycare centers, infectious bioaerosols, air pollutants

## Abstract

In this study, we investigated the concentration of airborne influenza virus in daycare centers and influencing factors, such as common cold prevalence, air pollutants, and meteorological factors. A total of 209 air samples were collected from daycare centers in Kaohsiung and the influenza virus was analyzed using real-time quantitative polymerase chain reaction. Air pollutants and metrological factors were measured using real-time monitoring equipment. Winter had the highest positive rates of airborne influenza virus and the highest prevalence of the common cold, followed by summer and autumn. The concentration of CO was significantly positively correlated with airborne influenza virus. Daycare center A, with natural ventilation and air condition systems, had a higher concentration of airborne influenza A virus, airborne fungi, and airborne bacteria, as well as a higher prevalence of the common cold, than daycare center B, with a mechanical ventilation system and air purifiers, while the concentrations of CO_2_, CO, and UFPs in daycare center A were lower than those in daycare center B. We successfully detected airborne influenza virus in daycare centers, demonstrating that aerosol sampling for influenza can provide novel epidemiological insights and inform the management of influenza in daycare centers.

## 1. Introduction

Children spend at least 8 h per day in daycare centers [1,2]. The adjusted odds ratios for the common cold, ear infection, and pneumonia in daycare centers relative to home care settings were 1.98, 2.34, and 1.79, respectively [3]. Moreover, the reported annual influenza attack rates for children (from 24% in 2016 to 47% in 2017) [4] were higher than the attack rates of SARS-CoV-2 (18.7%) [5]. Accordingly, studying the profiles of airborne influenza viruses in indoor environments, particularly in daycare centers, is crucial.

In addition to being a high-risk area for avian influenza virus, only six studies have detected airborne influenza viruses in indoor environments, of which one was conducted in an elementary school [6], one in a university [7], and four in hospitals [8,9,10,11] (Table 1). A longitudinal study conducted at a university reported that relative humidity, temperature, and ventilation influence the seasonal trends of influenza viruses; the study also indicated that the median influenza A and B virus concentrations were 20,000 and 14,696 copies/m^3^, respectively [7]. Another study revealed that the airborne influenza A virus-positive rate in an elementary school was 5% (5/96), with the virus concentration ranging from 0.2 to 19,000 copies/m^3^ [6]. Nevertheless, no study has investigated the concentration profiles of airborne influenza viruses in daycare centers and the influencing factors.

Children who attend daycare centers are exposed to higher levels of infectious agents and air pollutants—including carbon monoxide (CO), carbon dioxide (CO_2_), and ultrafine particles (UFPs)—than those who are cared for at home [12,13]. Studies have reported that children exposed to UFPs (aerodynamic diameter < 0.1 μm) have an increased risk of respiratory infection and hospital admission [14,15], and the amount of influenza virus found in the fraction of UFP_S_ less than 0.25 um was 15% [16]. Hence, we hypothesized that the concentration of UFPs could be a predictive factor for airborne influenza virus concentration. Poor ventilation along with high levels of CO_2_ in daycare centers causes the accumulation of indoor air pollutants and negatively affects health [17,18,19]. A study in Porto revealed that the concentrations of CO_2_ in nurseries and kindergartens were 1563 and 1492 ppm, respectively [20]. Scholars have also considered CO as an indicator of respiratory inflammation [21,22,23,24]. Specifically, studies have reported that patients with upper or lower respiratory tract infections and current smokers had increased concentrations of exhaled CO [21,22,23,24]. Therefore, investigating indoor CO_2_, UFPs, and CO was critical for discussing the influence factors when evaluating the airborne influenza virus in daycare centers.

Long-term exposure to airborne bacteria and fungi contributes to adverse health effects, such as irritation and asthma symptoms [25]. A study reported that concentrations of airborne bacteria and fungi in daycare centers in Taipei [26] were higher than those in Malaysia and Korea [27,28]. These differences can be attributed to the high temperature and relative humidity throughout the year in Taiwan. Therefore, the aim of the present study was to evaluate the concentration profiles of airborne influenza viruses, bacteria, and fungi, as well as CO, CO_2_, and UFPs in daycare centers. Factors influencing the concentration profiles, such as seasonal variation, meteorological factors, and ventilation type, including natural ventilation with air conditioning systems and mechanical ventilation systems with air purifiers, were also explored.

## 2. Methods

### 2.1. Air Sample Collection

In this study, we selected two daycare centers (denoted herein as daycare center A and daycare center B) with different ventilation systems in Kaohsiung City, Taiwan, for sample collection. Daycare center A is located near a busy road and has both a natural ventilation system and an air conditioning system. In autumn and winter, the windows are usually opened for ventilation without the air conditioning system. Daycare center B is located on a small lane and has both an air purifier and a mechanical exhaust system. Windows are rarely opened for ventilation throughout the year. Children attending daycare center B are typically required to wear masks whenever they are sick.

Data on the number of sick students (students who took medicine) and the total number of students in a class were collected through questionnaires on every sampling date. A 2-week sampling period was randomly chosen in every season in each daycare center. Air was continuously sampled from 9:00 am to 3:00 pm (6 h per day) for 10 consecutive working days. Air samples were not collected in daycare center A in spring because it was being renovated.

Air samplers were set up at a height of 1 m because this height was near the children’s breathing zone. Airborne influenza viruses were collected on 1.0 μm-pore polytetrafluoroethylene (Teflon; Pall Corporation, New York, NY, USA) membrane filters in disposable plastic cassettes at a sampling rate of 20 L/min for a 6-h sampling period (37 mm; HIBLOW SPP-25 GA, Techno Takatsuki, Osaka, Japan) [29,30,31]. The collected air samples were then transported at 4 °C to our laboratory and isolated within 3 days of collection. Viral RNA was stored at −80 °C and analyzed through quantitative real-time polymerase chain reaction (qPCR).

Duplicable airborne bacteria and airborne fungi were collected in the daycare centers by using portable microbiological air samplers (MAS-100; MERCK, Darmstadt, Germany). Before each sampling process, the air sampler was wiped with alcohol to avoid cross-contamination. The MAS-100 microbiological air samplers were loaded with tryptic soy agar (TSA; Difco Laboratories, Detroit, MI, USA) and malt extract agar (MEA; Difco Laboratories, Detroit, MI, USA) plates for bacteria and fungi, respectively. Other important information for treatment is described in the Supplementary File S1.

### 2.2. Air Pollutant and Environmental Factor Recording

CO, CO_2_, UFPs, temperature, relative humidity, and wind speed were monitored in real-time (Table 2), and the average concentration was calculated from the data collected on the sampling dates. All real-time monitoring instruments were calibrated at the beginning of this study and were annually recalibrated.

### 2.3. Reverse Transcription qPCR

Reverse transcription qPCR (RT-qPCR) was performed in accordance with the Minimum Information for Publication of Quantitative Real-Time PCR Experiments guidelines [32]. The amplification and detection processes were performed through an ABI PRISM 7500 Sequence Detection System. The primer and probe used for the amplification and detection of influenza A virus and influenza B virus are presented in Table S1. The specificity was 100% for both influenza A virus and influenza B virus [33]. The amplification and detection processes were performed through an ABI PRISM 7500 Sequence Detection System with the Taqman One-Step Reverse Transcriptase PCR Master Mix Reagents Kit (Applied Biosystems, Waltham, MA, USA) by using 5 μL of viral RNA solution in an end volume of 25 μL. All samples underwent qPCR assays in triplicate. The PCR mixture for influenza A virus and influenza B virus comprised 5 μL of RNA sample, 1 μL of primer (influenza A or B virus), 12.5 μL of 2× Master Mix without UNG, 0.625 μL of 40× MultiScribe and RNase Inhibitor Mix, 1 μL of TaqMan probe, and 4.875 μL of diethylpyrocarbonate water. The amplification and detection processes executed using the ABI PRISM 7500 Sequence Detection System (Applied Biosystems, Waltham, MA, USA) were performed under the following conditions: amplification at 48 °C for 10 min and at 95 °C for 10 min, followed by denaturation at 95 °C for 15 min, and then 40 cycles of annealing and extension at 60 °C for 1 min.

The sensitivity of the qPCR assays was examined in triplicate using 10-fold dilutions of plasmid cDNA, and the standard solution was diluted from 10^5^ to 10^0^. The threshold was set at 10 times the standard deviation of the mean baseline emission calculated for PCR cycles 3–10. The quantity of the product in a given field sample was determined using a standard curve of quantification cycle (C_q_) values in the same run. The calibration curve was linear over five orders of magnitude, with the R^2^ value being >0.990, the slope ranging between −3.3 and −3.4, and efficiency ranging between 96% and 100%. Trip blank and field blank controls were also evaluated for quality control in parallel, with no influenza virus RNA signal being used. Trip blank and field blank samples were evaluated for every 10 air samples, and side-by-side duplicate field samples yielded comparable results, with the relative difference being 9%.

The concentrations of airborne influenza viruses were calculated using the following equation: [detected concentration (copies/μL) × extracted volume (μL) × 1000 m^3^/L]/[sampling rate (L/min) × sampling duration (min)].

The limit of detection (LOD) for influenza A virus was 30 copies/reaction, and that for influenza B virus was 19.5 copies/reaction. Moreover, the LOD for airborne influenza A virus was 66.7 copies/m^3^, and that for airborne influenza B virus was 43.3 copies/m^3^. The standard curve (positive controls) and negative controls were evaluated in triplicate in each run, and the data were included only when the negative control was identified to have no signal.

### 2.4. Statistical Analysis

All data were analyzed using IBM SPSS (Version 20, SPSS Institute, Chicago, IL, USA) and SAS (Version 9.4, SAS Institute, Cary, NC, USA). The data collected in spring were excluded from the analysis because of the renovation of daycare center A during the sampling period. The data were presented as a non-normal distribution determined by a one-sample Kolmogorov–Smirnov test. We assumed that not detected (N.D) was 0.1 copies/m^3^ of the samples for calculating the geometric mean (GM) and geometric standard deviation due to the fact that the positive rates of airborne influenza A and B virus were 30% and 58%, respectively. A chi-square test was used to determine the seasonal variations of the positive rates of the influenza viruses in the daycare centers. The differences between daycare center A and daycare center B were estimated using the Mann–Whitney U test. The Kruskal–Wallis test was used to estimate seasonal differences, and the post hoc test was used for further analysis. A *p*-value of <0.05 was considered significant. The association between bioaerosols, meteorological factors, and air pollutants was evaluated using Spearman’s rank correlation. To address the problem of multiple comparisons, we used the Benjamini–Hochberg false discovery rate (FDR) to reduce the risk of type I errors [34,35,36], and an FDR of <0.05 was considered statistically significant for Spearman’s rank correlation results. Multivariate linear regression was used to investigate the association between the airborne influenza viruses and other influencing factors, and a log10 transformation was performed for bioaerosols, meteorological factors, air pollutants, and common cold prevalence.

## 3. Results

Table 3 presents descriptive statistics regarding the measured bioaerosols in the daycare centers. A total of 209 air samples were collected in the daycare centers. The overall positive rate of the influenza A virus was 30% (53/179), and that of the influenza B virus was 58% (103/179). The GM concentrations of airborne influenza A virus, airborne influenza B virus, airborne bacteria, and airborne fungi were 4.6 copies/m^3^, 2.5 copies/m^3^, 4000 CFU/m^3^, and 830 CFU/m^3^, respectively. The GM concentrations of both airborne influenza A virus and influenza B virus were higher in daycare center A (8.6 and 3.4 copies/m^3^, respectively) than in daycare center B (2.5 and 1.8 copies/m^3^, respectively). The GM concentrations of airborne bacterial and fungal bioaerosols were significantly higher in daycare center A (5000 and 1500 CFU/m^3^, respectively) than in daycare center B (3200 and 480 CFU/m^3^, respectively). The prevalence of the common cold, calculated as the number of sick students divided by the number of total students in class, was significantly higher in daycare center A (GM, 23.2%) than in daycare center B (GM, 16.9%).

Table 4 lists descriptive statistics regarding the measured air pollutants and meteorological factors in the daycare centers. The GM concentrations of CO, CO_2_, and UFPs were significantly higher in daycare center B (4.4 ppm, 1257 ppm, and 3.9 × 10^10^ pt/cc, respectively) than in daycare center A (3.1 ppm, 585 ppm, and 2.6 × 10^10^ pt/cc, respectively). In addition, the GM of wind speed in daycare center A (0.10 m/s) was higher than that in daycare center B (0.07 m/s).

Table 5 presents the concentrations of the bioaerosols, concentrations of the air pollutants, and levels of the meteorological factors in summer, autumn, and winter in the daycare centers. The percentages of airborne bacteria, airborne fungi, CO, and CO_2_ were determined to exceed the acceptable levels specified in the Indoor Air Quality Act established by the Environmental Protection Administration, Executive Yuan, ROC, Taiwan [37] by 100%, 58%, 93%, and 76%, respectively. We observed significant seasonal differences in the positive rates of airborne influenza virus and in the prevalence of the common cold. Winter had the highest positive rates and GM concentrations of airborne influenza A virus (52% and 88.4 copies/m^3^, respectively) and airborne influenza B virus (45% and 12.9 copies/m^3^, respectively), followed by summer and autumn. Moreover, winter had the highest prevalence of the common cold (24.1%), followed by autumn (18.0%) and summer (17.8%). The GM concentration of CO in winter (6.1 ppm) was significantly higher than those in summer (2.8 ppm) and autumn (2.9 ppm). The GM temperature in summer was 27.3 °C, which was significantly higher than those in autumn (25.6 °C) and winter (21.8 °C; *p* < 0.001). The relative humidity level in autumn (73.1%) was significantly higher than those in winter (70.5%) and summer (65.3%) (*p* < 0.001). We observed that wind speed in summer (0.15 m/s) was significantly higher than those in autumn (0.08 m/s) and winter (0.06 m/s; *p* < 0.001).

Table 6 presents the correlation between bioaerosols, air pollutants, and meteorological factors. The concentration of airborne influenza A virus had a significant positive correlation with the positive rate of airborne influenza A virus (correlation coefficient, r = 0.97, FDR = 0.001), and the concentration of airborne influenza B virus had a significant positive correlation with the positive rate of airborne influenza B virus (r = 0.79, FDR = 0.001). We observed a significant positive correlation between the prevalence of the common cold and the concentrations of both airborne influenza A virus (r = 0.36, FDR = 0.027) and airborne fungi (r = 0.47, FDR = 0.002). Both airborne influenza A virus and airborne influenza B virus exhibited a negative correlation with temperature (FDR < 0.05). The positive rates and concentrations of both airborne influenza A virus and airborne influenza B virus were significantly and positively correlated with the concentration of CO (FDR < 0.05).

Multivariate linear regression was used to evaluate the association between the airborne influenza viruses and environmental factors after adjustment for temperature, relative humidity, and wind speed (Table 7). Airborne influenza A virus was significantly associated with the prevalence of the common cold (standardized beta coefficient [β] = 5.86, *p* = 0.017) and CO (β = 4.45, *p* = 0.036). Airborne influenza B virus was also significantly associated with CO (β = 3.34, *p* = 0.046) and UFPs (β = 4.02, *p* < 0.001).

## 4. Discussion

Although [38] investigated DNA and RNA viral bioaerosols in daycare centers, they did not mention airborne influenza viruses; this is the first study to describe the concentration profiles of airborne influenza viruses in daycare centers in the real world. To date, only six studies have tested for airborne influenza viruses in hospitals and schools (Table 1). The positive rates of airborne influenza A virus and airborne influenza B virus (30% and 58%, respectively) in our observation are considerably higher than those observed in schools (5% for airborne influenza A virus in elementary school [6]; 17% and 5% for airborne influenza A virus and B virus in university [7]). The median concentrations of positive samples of airborne influenza A virus and airborne influenza B virus in a university were 20,400 and 14,696 copies/m^3^ [7], respectively, which are considerably higher than those observed in our study (10,200 and 8.35 copies/m^3^ for airborne influenza A virus and airborne influenza B virus). Possible reasons are that, during the sampling period (2016–2018) in the university, an influenza epidemic occurred in Hong Kong [7]. They collected their air samples for shorter periods (30 min at 3.5 L/min) in canteens, lecture halls, shuttle buses, and the University Health Service during peak hours of human flow. The corresponding LOD was 8163 copies/m^3^ in a previous study [7], which is higher than those used in the present study. In elementary school, the median concentration of positive samples of airborne influenza A virus concentration was 3800 copies/m^3^ [6], which is lower than that observed in our study. Accordingly, the high positive rates and concentrations of airborne influenza viruses around susceptible young children in daycare centers pose a critical public health problem that warrants addressing.

Studies have reported annual influenza virus outbreaks in winter [8,9,10,11], which is consistent with our results, revealing positive rates and concentrations of both airborne influenza A virus and airborne influenza B virus. Our results reveal a significant negative correlation between airborne influenza viruses and temperature, consistent with the findings of studies on both animals and humans [8,39,40,41]. Animal studies have reported that low-temperature conditions facilitated aerosol transmission in guinea pig models [40,41]. Furthermore, an epidemiological study found a negative association between influenza viruses and temperature and found that weekly mean temperature had a significant negative association with weekly numbers of outpatients with influenza A virus and influenza B virus [39]. The relative risks of influenza A virus increased when temperatures were below 15 or above 20 °C [39]. Another study also observed the effect of temperature and relative humidity on positive samples of airborne influenza A virus in hospitals [8].

The present study revealed a positive correlation between airborne influenza A virus and the prevalence of the common cold; this study also indicated the highest prevalence occurred in winter. When the numbers of sick children increase in daycare centers, people should consider indoor air quality, particularly with regard to airborne influenza viruses. We noted that the prevalence of the common cold and the concentration of bioaerosols, including airborne influenza viruses, airborne bacteria, and airborne fungi, in daycare center A was significantly higher than that in daycare center B. A possible reason for this difference is that daycare center B typically requires children with respiratory symptoms to wear masks and use air purifiers. Mask-wearing among children could reduce respiratory infection rates [42,43]. In a laboratory study, using air purifiers in wards under standard conditions could achieve a 100% purification rate for influenza viruses after 30 min [44]. Additionally, air purifiers with high-efficiency particulate air (HEPA) filtration systems could reduce the concentration of airborne fungi, with efficiency rates of 32% and 99% in households in the United States and Japan, respectively [45,46].

Our results reveal that the mean concentrations of airborne bacteria and fungi were 4700 and 1100 CFU/m^3^, respectively, which are higher than those reported by studies conducted in other areas such as Malaysia (754.8 and 699.7 CFU/m^3^ for bacteria and fungi, respectively), Korea (418 CFU/m^3^ for bacterial and fungal bioaerosols), and Taipei (735 and 1212 CFU/m^3^ for bacteria and fungi, respectively) [26,27,28]. The present study also revealed that the percentages of airborne bacteria and airborne fungi exceeded acceptable levels (1500 CFU/m^3^ for airborne bacteria and 1000 CFU/m^3^ for airborne fungi) by 100% and 58%, respectively, which are considerably higher concentrations than those observed in daycare centers in Malaysia, in which the percentages of airborne bacteria and airborne fungi exceeded the acceptable levels recommended by the National Institute of Occupational Safety and Health (500 CFU/m^3^ for airborne bacteria and 1000 CFU/m^3^ for airborne fungi) by 50% and 20%, respectively [28]. In Taiwan, the temperature and relative humidity are high throughout the year, and the climate may be favorable for the breeding and transmission of bioaerosols in microenvironments [47].

The concentrations of CO_2_, CO, and UFPs in daycare center B were higher than those in daycare center A. A previous study considered CO_2_ concentration as an indicator of ventilation and indicated that poor ventilation may lead to the accumulation of air pollutants in indoor environments [19]. Daycare center A uses natural ventilation and air conditioning systems with open windows in autumn and winter. Daycare center B uses both a mechanical exhaust system and an air purifier throughout the year and rarely opens the windows. This may explain why daycare center B exhibited higher concentrations of CO_2_. Although the use of air purifiers with HEPA filters can reduce UFPs in households by approximately 30–47% [45,48], the concentration of UFPs in daycare center B was higher than that in daycare center A. Daycare center B uses combustion appliances in preparing lunch. Previous studies have indicated that the use of combustion appliances for food preparation could increase the concentrations of UFPs and CO [49,50]. This may be why the UFP concentration was higher in daycare center B. We observed that both airborne influenza A virus and airborne influenza B virus exhibited a significant positive correlation with CO, and our multiple linear regression adjusted for temperature, relative humidity, and wind speed also revealed similar results. Previous studies have reported that patients with upper and lower respiratory tract infections exhibited increased concentrations of exhaled CO [21,22]. This may explain the positive correlation between airborne influenza viruses and CO in the daycare centers in this study. Exhaled CO should be investigated further in future studies.

Some limitations of this study must be mentioned. First, we considered CO as an indicator of respiratory infection, and we observed a positive correlation between airborne influenza viruses and CO; however, we monitored only indoor CO in the daycare centers and not exhaled CO from children. Second, we selected only two daycare centers, which were not representative of all daycare centers. Third, daycare center A was being renovated during the spring of our sampling period; therefore, we could not obtain the concentrations of airborne influenza viruses in daycare center A in spring, which meant we could not present the profile of airborne influenza viruses over the whole years in daycare centers. As such, the comparisons between daycare centers A and B are only based on information from summer, fall, and winter.

## 5. Conclusions

The concentrations of airborne influenza viruses, bacteria, and fungi in daycare center A with natural ventilation and air conditioning systems were significantly higher than those in daycare center B with a mechanical ventilation system, an air purifier, and mask-wearing among sick children. The prevalence of the common cold was highest in winter. A significant positive correlation was observed between the airborne influenza viruses and CO.

## 6. Environmental Implication

This is the first study describing the concentration of airborne influenza virus and influencing factors in daycare centers. Common cold prevalence was positively correlated with airborne influenza virus concentration. The DCC with natural ventilation and air conditioning had higher bioaerosol levels, whereas the DCC with mechanical ventilation and air purifiers had lower air pollutant levels.

## Figures and Tables

**Table 1:** Positive rates, concentrations, and other information regarding air samples of influenza viruses.

Reference	Sampling Location	Sampling Collection	Positive Rate	Concentration	Limit of Detection
Our study	Taiwan; daycare center	Samplers: 1.0-μm-pore Teflon membrane filters in disposable plastic cassettes with HIBLOW SPP-25 GA (Techno Takatsuki, Osaka, Japan) Sampling rates and time: 20 L/min for 6 h Sampling periods: August 2006 to May 2007	IAV, 30% (53/179) IBV, 58% (103/179)	Median concentration of positive samples: 10,200 and 8.35 copies/m^3^ of IAV and IBV, respectively	IAV: 30 copies/reaction (66.7 copies/m^3^ air) IBV: 19.5 copies/reaction (43.3 copies/m^3^ air)
[6]	USA; elementary school	Samplers: SKC AirChek XR5000 pump (SKC, Eighty Four, PA, USA) Sampling rates and time: 3.5 L/min for 4 h, and four times per week Sampling periods: an 8-week sampling period (February–March)	5% (5/96)	Median concentration of positive samples: 3800 copies/m^3^	1 copy/reaction (29.8 copies/m^3^ air)
[7]	China; university (canteens, lecture halls, shuttle buses, and the University Health Service)	Samplers: National Institute for Occupational Safety and Health (NIOSH) bioaerosol sampler (BC251) with SKC AirChek XR5000 pump (SKC, Eighty Four, PA, USA) Sampling rates and time: 3.5 L/min for 30 min, and 6–13 air samplers per week. Sampling periods: October 2016 to June 2018 except during the summer holidays in 2017 (weeks 19–35) and on public holidays	IAV, 17% (117/695); IBV, 5% (31/694)	Median concentration of positive samples: 20,400 and 14,696 copies/m^3^ of IAV and IBV, respectively	2 copies/reaction (8163 copies/m^3^ air).
[8]	China; fever clinic, child ward, and adult ward with patients with confirmed influenza	Samplers: Bio-Capturer (Bio- enrichment Technology, Hangzhou, China) Sampling rates and time: 40 L/min for 4 h Sampling periods: two 7-day periods of 23–29 January 2018 (peak flu activity) and 9–15 April 2018 (low flu activity)	fever clinic IAV, 14% (1/7); IBV, 14% (1/7)	-	-
			child ward IAV, 14% (3/21); IBV, 19% (4/21)	-	-
			adult ward IAV, 0% (0/7); IBV, 14% (1/7)	-	-
[9]	USA; urgent care clinic (patient waiting room, examination rooms, procedure rooms)	Samplers: Stationary NIOSH 2-stage cyclone aerosol samplers Sampling rates and time: 4–5 h Sampling periods: 11 days during February 2009.	IAV, 17% (46/264); IBV, 1% (3/264)	0.1–7.3 pg/m^3^	-
[10]	Hong Kong; patient room with patients confirmed influenza A virus	Samplers: NIOSH samplers Sampling rates and time: 3.5 L/min for 4 h Sampling periods: during the winter influenza season of 2014–2015.	50% (5/10)	162 and 144 copies/m^3^ of >4 μm and 1–4 μm, respectively	208 copies/mL
[11]	USA; emergency department (waiting room, children’s waiting room, examination rooms)	Samplers: NIOSH 2-stage cyclone aerosol sampler Sampling rates and time: 3.5 L/min for 4–5 h. Sampling periods: 6 afternoons during February 2008	53%	-	-

IAV, influenza A virus; IBV, influenza B virus.

**Table 2:** Concentration range, precision, and accuracy of sampling instruments.

	Range	Precision	Accuracy	Sampling Instrument
CO_2_	0 to 5000 ppm	1 ppm	±50 ppm	Model 8760 IAQ-CALC Meter, TSI, Shoreview, MN, USA
CO	0 to 500 ppm	0.1 ppm	±3 ppm	
Temperature	0–60 °C	0.1 °C	±0.6 °C	
RH	5–95%	0.1%	±3.0%	
Wind speed	0 to 20.00 m/s	-	±0.025 m/s	VelociCheck (Model 8330, TSI, Shoreview, MN, USA)
UFPs	0 to 500,000 pt/cc	-	-	P-TRAK Ultrafine Particle Counter (Model 8525, TSI, Shoreview, MN, USA)

**Table 3:** Descriptive statistics regarding bioaerosols in daycare centers in summer, autumn, and winter.

		Daycare Center A (*n* = 89)	Daycare Center B (*n* = 90)	*p*-Value	All (*n* = 179)
Influenza A virus (copies/m^3^)	Positive rate	34% (30/89)	33% (30/90)	0.861	30% (53/179)
	Mean (SD)	1.6 × 10^5^ (7.8 × 10^5^)	5.9 × 10^3^ (2.8 × 10^4^)	0.561	8.0 × 10^4^ (5.5 × 10^5^)
	GM (GSD)	8.6 (10.2)	2.5 (2.1)		4.6 (3.3)
	Median	0.10	0.10		0.1
	Range	0.10~4.2 × 10^6^	0.10~1.5 × 10^5^		0.1~4.2 × 10^6^
Influenza B virus (copies/m^3^)	Positive rate	74% (66/89)	70% (63/90)	0.436	58% (103/179)
	Mean (SD)	1.2 × 10^4^ (6.2 × 10^4^)	3.0 × 10^5^ (1.1 × 10^6^)	0.849	1.6 × 10^5^ (8.2 × 10^5^)
	GM (GSD)	3.4 (2.2)	1.8 (2.1)		2.5 (1.7)
	Median	1.7	2.7		2.6
	Range	N.D.~3.3 × 10^5^	0.1~4.6 × 10^6^		0.1~4.6 × 10^6^
Bacteria (CFU/m^3^)	Mean (SD)	5.8 × 10^3^ (3.6 × 10^3^)	3.5 × 10^3^ (1.8 × 10^3^)	0.006 *	4.7 × 10^3^ (3.1 × 10^3^)
	GM (GSD)	5.0 × 10^3^ (5.2 × 10^2^)	3.2 × 10^3^ (3.0 × 10^2^)		4.0 × 10^3^ (3.0 × 10^2^)
	Median	4.7 × 10^3^	3.4 × 10^3^		3.7 × 10^3^
	Range	1.3 × 10^3^~1.7 × 10^4^	1.2 × 10^3^~9.9 × 10^3^		1.2 × 10^3^~1.7 × 10^4^
Fungi (CFU/m^3^)	Mean (SD)	1.6 × 10^3^ (7.6 × 10^2^)	5.6 × 10^2^ (5.2 × 10^2^)	<0.001 *	1.1 × 10^3^ (8.2 × 10^2^)
	GM (GSD)	1.5 × 10^3^ (1.1 × 10^2^)	4.8 ×10^2^ (6.6 × 10^1^)		8.3 × 10^2^ (9.0 × 10^1^)
	Median	1.2 × 10^3^	3.3 × 10^2^		1.0 × 10^3^
	Range	9.3 × 10^2^~4.1 × 10^3^	1.6 × 10^2^~2.7 × 10^3^		1.6 × 10^2^~4.1 × 10^3^
Prevalence of common cold (%)	Mean (SD)	24.0 (6.2)	17.7 (5.9)	<0.001 *	20.8 (6.8)
	GM (GSD)	23.2 (1.2)	16.9 (0.9)		19.8 (0.8)
	Median	23.3	17.8		19.2
	Range	13.5~35.4	9.4~42.0		9.4~42.0

Mann–Whitney U test. SD, standard deviation. GM, geometric mean; GSD, geometric standard deviation; * *p* < 0.05, N.D., not detected.

**Table 4:** Descriptive statistics regarding air pollutants and meteorological factors in daycare centers in summer, autumn, and winter.

		Daycare Center A (*n* = 89)	Daycare Center B (*n* = 90)	*p*-Value	All (*n* = 179)
CO (ppm)	Mean (SD)	3.5 (1.6)	5.1 (1.8)	0.004 *	4.1 (1.8)
	GM (GSD)	3.1 (0.3)	4.4 (0.3)		3.7 (0.2)
	Median	3.1	5.6		3.5
	Range	1.3~5.9	2.7~8.7		1.3~8.7
CO_2_ (ppm)	Mean (SD)	606 (160)	1300 (211)	<0.001 *	946 (384)
	GM (GSD)	585 (29)	1257 (39)		863 (50)
	Median	612	1320		898
	Range	344~1010	822~1667		344~1661
Ultrafine particles (pt/cc)	Mean (SD)	2.8 × 10^10^ (9.9 × 10^9^)	5.6 × 10^10^ (6.6 × 10^10^)	0.002 *	4.0 × 10^10^ (4.8 × 10^10^)
	GM (GSD)	2.6 × 10^10^ (1.9 × 10^9^)	3.9 × 10^10^ (4.2 × 10^9^)		3.2 × 10^10^ (2.2 × 10^9^)
	Median	2.9 × 10^10^	4.0 × 10^10^		3.2 × 10^10^
	Range	1.0 × 10^10^~4.8 × 10^10^	2.3 × 10^9^~3.9 × 10^11^		1.0 × 10^10^~3.9 × 10^11^
Temperature (°C)	Mean (SD)	25.2 (3.9)	24.8 (1.0)	0.070	24.9 (2.9)
	GM (GSD)	24.9 (0.8)	24.6 (0.2)		24.7 (0.4)
	Median	26.4	24.9		24.9
	Range	17.6~30.5	22.5~26.9		17.6~30.5
Relative humidity (%)	Mean (SD)	70.2 (4.9)	66.7 (8.2)	0.850	69.9 (6.0)
	GM (GSD)	59.7 (6.6)	69.4 (1.3)		64.4 (3.6)
	Median	69.0	65.9		69.1
	Range	60.9~78.6	51.9~84.0		56.0~84.0
Wind speed (m/s)	Mean (SD)	0.13 (0.07)	0.09 (0.04)	0.033 *	0.11 (0.06)
	GM (GSD)	0.10 (0.01)	0.07 (0.01)		0.09 (0.01)
	Median	0.12	0.08		0.11
	Range	0.01~0.29	0.01~0.17		0.01~0.30

Mann–Whitney U test. SD, standard deviation. GM, geometric mean; GSD, geometric standard deviation; * *p* < 0.05.

**Table 5:** Concentrations of bioaerosols, meteorological factors, and air pollutants in summer, autumn, and winter in daycare centers.

	GM (GSD), [Exceeding Acceptable Levels %]			
	Summer (*n* = 59)	Autumn (*n* = 60)	Winter (*n* = 60)	*p*-Value
Bioaerosol				
PR of Influenza A virus (%)	47	5	55	0.002 *
PR of Influenza B virus (%)	32	85	95	<0.001 *
Influenza A virus (copies/m^3^)	6.0 (7.0)	0.2 (0.1)	88.4 (126)	0.002 ^c,^*
Influenza B virus (copies/m^3^)	0.3 (0.5)	3.7 (2.2)	12.9 (9.7)	0.001 ^a,b,^*
Bacteria (CFU/m^3^)	4.2 × 10^3^ (6.3 × 10^2^), [100%]	4.5 × 10^3^ (5.1 × 10^2^), [100%]	3.4 × 10^3^ (4.2 × 10^2^), [100%]	0.263
Fungi (CFU/m^3^)	5.6 × 10^2^ (1.1 × 10^2^), [58%]	9.7 × 10^2^ (1.9 × 10^2^), [55%]	1.0 × 10^3^ (1.4 × 10^2^), [60%]	0.086
Prevalence of common cold (%)	17.8 (1.2)	18.0 (0.9)	24.1 (1.9)	0.001 ^b,c,^*
Air pollutants				
CO (ppm)	2.8 (0.2), [84%]	2.9 (0.2), [90%]	6.1 (0.3), [100%]	<0.001 ^b,c,^*
CO_2_ (ppm)	812 (73), [78%]	760 (90), [50%]	1040 (81), [100%]	0.085
Ultrafine particles (pt/cc)	2.6 × 10^10^ (2.9 × 10^9^)	2.9 × 10^10^ (2.6 × 10^9^)	4.4 × 10^10^ (5.6 × 10^9^)	0.113
Meteorological factors				
Temperature (°C)	27.3 (0.4)	25.6 (0.2)	21.8 (0.5)	<0.001 ^a,b,c,^*
Relative humidity (%)	65.3 (1.2)	73.1 (1.3)	70.5 (1.0)	<0.001 ^a,b,c,^*
Wind speed (m/s)	0.15 (0.01)	0.08 (0.01)	0.06 (0.01)	<0.001 ^a,b,^*

GM, geometric mean; GSD, geometric standard deviation; the positive rate (PR) calculation used the chi-square tests for analyzing the difference, and, for the other variables, the Kruskal–Wallis Test was used to analyze the difference. Post-hoc test: ^a^ summer vs. autumn *p* < 0.05; ^b^ summer vs. winter, *p* < 0.05; ^c^ autumn vs. winter, *p* < 0.05. * *p* < 0.05.

**Table 6:** Correlations between bioaerosols and factors including air pollutants and meteorological factors in daycare centers.

	PR of Influenza A Virus (%)	PR of Influenza B Virus (%)	Influenza A Virus (copies/m^3^)	Influenza B Virus (copies/m^3^)	Bacteria (CFU/m^3^)	Fungi (CFU/m^3^)
PR of influenza A virus (%)	1.000					
PR of influenza B virus (%)	0.004	1.000				
Influenza A virus (copies/m^3^)	0.969 **	0.053	1.000			
Influenza B virus (copies/m^3^)	0.047	0.794 **	0.107	1.000		
Bacteria (CFU/m^3^)	−0.135	0.266	−0.085	0.185	1.000	
Fungi (CFU/m^3^)	−0.129	0.296	0.009	0.232	0.558 **	1.000
Prevalence of common cold (%)	0.258	0.159	0.363 *	0.171	0.263	0.469 **
CO (ppm)	0.420 **	0.378 *	0.421 **	0.408 *	−0.232	−0.173
CO_2_ (ppm)	0.116	0.090	0.070	0.119	−0.309	−0.531 **
Ultrafine particles (pt/cc)	0.249	0.294	0.230	0.278	−0.252	−0.249
Temperature (°C)	−0.318	−0.392 *	−0.357 *	−0.331 *	0.084	0.011
Relative humidity (%)	−0.138	0.306	−0.065	0.268	0.193	0.127
Wind speed (m/s)	−0.008	−0.268	−0.050	−0.119	0.147	0.020

Spearman correlation. PR, positive rate; * false discovery rate (FDR) < 0.05; ** FDR < 0.01.

**Table 7:** Multivariate linear regression analysis results regarding correlation of factors, including bioaerosols, air pollutants, and meteorological factors, with airborne influenza viruses.

	Variables	Influenza A Virus		Influenza B Virus	
Factors		β (95% CI)	*p*-Value	β (95% CI)	*p*-Value
Bioaerosols					
Influenza A virus				−0.06 (−0.27, 0.16)	0.584
Influenza B virus		−0.09 (−0.44, 0.25)	0.584		
Bacteria		−0.55 (−3.03, 1.92)	0.656	0.58 (−1.37, 2.54)	0.554
Fungi		−0.07 (−1.82, 1.67)	0.933	0.78 (−0.59, 2.15)	0.258
Prevalence of common cold		5.86 (1.12, 10.61)	0.017 *	0.03 (−3.96, 4.01)	0.989
Air pollutants					
CO		4.45 (0.31, 8.60)	0.036 *	3.34 (0.06, 6.63)	0.046 *
CO_2_		−1.09 (−4.66, 2.48)	0.544	0.82 (−2.00, 3.65)	0.561
Ultrafine particles		−0.12 (−3.00, 2.77)	0.936	4.02 (2.02, 6.02)	<0.001 *

Multivariate linear regression was adjusted for temperature, relative humidity, and wind speed, and all of the variables were log10 transformed. * *p* < 0.05.

## Data Availability

Data will be available if required.

## Supplementary Materials

The following supporting information can be downloaded at: https://www.mdpi.com/article/10.3390/v16060822/s1, File S1: Materials and methods; Table S1: Primer and probe used for the amplification and detection of influenza A virus and influenza B virus. References [30,33,51,52] are cited in the Supplementary Materials.

## Abbreviations

CO	carbon monoxide
CO_2_	carbon dioxide
UFPs	ultrafine particles
ppm	parts per million
qPCR	quantitative real-time polymerase chain reaction
RT-qPCR	reverse transcription qPCR
LOD	limit of detection
N.D	not detected
GM	geometric mean
FDR	false discovery rate
