# Supplementary material for: Airborne Influenza Virus in Daycare Centers
Source: Viruses. 2024 May 22;16(6):822. doi: 10.3390/v16060822 (PMC11209538; doi:10.3390/v16060822)
Supplement: Supplementary file 1 [file viruses-16-00822-s001.zip › viruses-3001725-supplementary.pdf]

File S1. Materials and method.

Air sample collection and RNA isolation

Before sampling, filters and support pads were autoclaved, and plastic cassettes were sterilized with ethylene oxide. RNA isolation was executed using commercially available QIAamp Viral RNA Mini Kits (Qiagen GmbH, Hilden, Germany). The isolation procedure was performed in accordance with the manufacturer's recommendation, except that in our study, the sampled Teflon filter was folded into quadrants with the virus inside and then placed upside down into the buffer AVL–carrier RNA in a 1.5-mL microcentrifuge tube [1].

Airborne bacteria and fungi

After transportation to the laboratory, the TSA plates were placed in an inverted position and incubated at 37 °C for 24 h, and the MEA plates were incubated at 25 °C; the colonies were counted after 48 h [2]. The actual colony count per each culture plate was corrected using the positive hole correction table from Operator's manual MAS-100 [3]. The concentrations of airborne bacterial and fungal bioaerosols are expressed herein as colony-forming units per cubic meter (CFU/m^3).

Table S1. Primer and probe used for the amplification and detection of influenza A virus and influenza B virus.

**Type of virus**		**primer**	**probe**	**Reference**
Influ-A (M gene)	Forward	5′ GGACTGCAGCGGAGTCGCTT	5′FAM-CTCAGTAATTGTGCTGGTG-CACCTGCCA-TAMRA	[4]
	Reverse	5′ CATCCTGTTGGATATGTGGCCCAT<br>5′ CATTCTGTTGGATATGTGGCCCAT		
Influ-B (HA gene)	Forward	5′ AAATACGGTGGATTAAATAAAA-GCAA	5′FAM-CACCCATATTGCG-CAAATTCCTATGGC-TAMRA	
	Reverse	5′ CCAGCAATAGCTCCGAAGAAA		

Reference

1. Chen, P.S.; Tsai, F.T.; Lin, C.K.; Yang, C.Y.; Chan, C.C.; Young, C.Y.; Lee, C.H. Ambient influenza and avian influenza virus during dust storm days and background days. *Environ. Health Perspect.* **2010**, *118*, 1211–1216. https://doi.org/10.1289/ehp.0901782.
2. Chi, M.-C.; Li, C.-S. Fluorochrome in monitoring atmospheric bioaerosols and correlations with meteorological factors and air pollutants. *Aerosol Science and Technology*. **2007**, *41*, 672–678. https://doi.org/10.1080/02786820701383181
3. Stec, J; Lenart-Boroń A. Assessment of microbiological aerosol concentration in selected healthcare facilities in southern Poland. *Cent Eur J Public Health*. **2019**, *27*, 239–244. 10.21101/cejph.a5681
4. van Elden, L.J.; Nijhuis, M.; Schipper, P.; Schuurman, R.; van Loon, A.M. Simultaneous detection of influenza viruses A and B using real-time quantitative PCR. *J. Clin. Microbiol.* **2001**, *39*, 196–200. https://doi.org/10.1128/jcm.39.1.196-200.2001.
